# Role of Exosomal miR‐223 in Chronic Skeletal Muscle Inflammation

**DOI:** 10.1111/os.13232

**Published:** 2022-03-16

**Authors:** Yuan Tian, Tie‐shan Wang, He Bu, Guo Shao, Wei Zhang, Li Zhang

**Affiliations:** ^1^ Department of Acupuncture‐Moxibustion and Tuina Beijing University of Chinese Medicine Beijing China; ^2^ Department of Acupuncture‐Moxibustion and Tuina The Second Affiliated Hospital of Baotou Medical College Baotou China; ^3^ Beijing Research Institute of Chinese Medicine Beijing University of Chinese Medicine Beijing China; ^4^ Center for Translational Medicine and Department of Laboratory Medicine the Third People's Hospital of Longgang District Shenzhen China; ^5^ Department of Pathology the First Affiliated Hospital of Baotou Medical College Baotou Inner Mongolia China

**Keywords:** Chronic inflammation, Exosome, miR‐223, Skeletal muscle injury, Targeted therapy

## Abstract

As skeletal muscle is one of the largest organs in the body, its damage can directly reflect a decline in somatic function, thus, further affecting daily life and health. Inflammation is a prerequisite for the repair of injured skeletal muscles. Chronic inflammation induced by inadequate repair in skeletal muscle aggravates tissue injury. Exosomes regulate inflammatory responses to facilitate the repair of skeletal muscle injury. Moreover, exosomal miR‐223 with high specificity is the most abundant miRNA in peripheral blood and regarded as biomarkers for inflammation post skeletal muscle injury, which warrants further investigation. Available studies have demonstrated that exosomal miR‐223 negatively correlates with TNF‐α levels in serum and regulates the canonical inflammatory NF‐κB signaling pathway. miR‐223 is a negative feedback regulator with great potential for adjusting inflammatory imbalance and promoting skeletal muscle repair. The research on the regulation of negative feedback factors in the inflammatory signaling pathway is essential in biology and medicine. Therefore, this review mainly elaborates the formation, heterogeneity and markers of exosomes and points out exosomal miR‐223 as a beneficial role in chronic skeletal muscle inflammation and can be expected to be a potential therapeutic target for skeletal muscle damage.

## Graphical abstract

**Fig 1. Biogenesis and identification of exosomes.** Identification of ectosomes and exosomes. (A) Endocytosis and plasma membrane invagination. (B) Formation of early sorting endosomes (ESEs). (C) ESEs give rise to late sorting endosomes(LSEs). (D) LSEs are gradually matured and transformed into multivesicular bodies (MVBs) containing exosomes. (E) MVBs are degraded by lysosomes. (F) The release of exosomes.

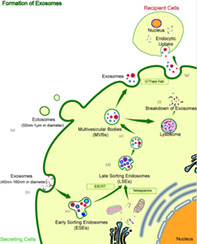

## Introduction

As one of the largest organs in the human body^1^, ^2^, skeletal muscle plays a key role in exercise^3^, breathing^4^, and metabolism^5^, participating in many vitalphysiological functions of the living body^6^, ^7^. Generally, 50% of the cases of acute injury are transformed into chronic due to the limitation of diagnosis and treatment and long‐term negligence of the harmfulness of skeletal muscle inflammation. For instance, chronic inflammation after lumbar multifidus muscle injury could result in recurrent low back pain, which is refractory for a long time^8^, ^9^. Therefore, it is one of the clinical problems that chronic inflammation after skeletal muscle injury demands urgent attention. Current studies have unraveled exosomes correlate with inflammatory diseases^10^, ^11^ and have potential advantages in the targeted therapy of chronic inflammation after muscle injury^12^ to emerge as a state‐of‐the‐art therapeutic strategy for treating chronic inflammation of skeletal muscle^13^, ^14^.

Most cells secrete exosomes, and the functions of exosomes depend on the type of cell from which they originate^15^. Exosomes contain specific microRNAs (miRNAs), proteins, and other biologically active substances, which have great potential to be applied as non‐invasive markers of diseases^16^, ^17^. Patients with different diseases release exosomes containing specific RNAs and proteins into the circulation^18^, ^19^, ^20^. An existing study has demonstrated that miR‐223 is the most abundant miRNA in the microvesicles isolated from peripheral blood of healthy donors^21^. These microvesicles may be originated from peripheral blood mononuclear cells which are the main source of circulating exosomes^22^. Furthermore, several studies have substantiated the implications of exosomal miR‐223 in the inflammatory response to skeletal muscle injury^17^, ^23^, ^24^. It has been reported that miR‐223 can down‐regulate TNF‐α and other pro‐inflammatory factors^25^, ^26^, ^27^, suppresses inflammatory infiltration, and reduce the area of necrotic muscle tissues^28^. Therefore, miR‐223 may be a potential biomarker and therapeutic target in chronic inflammation related to insufficient regeneration and repair following muscle injury, although which warrants further verification.

## Exosomes

### Formation of Exosomes

Since 2011, the International Society for Extracellular Vesicles (ISEV: www.isev.org/) has paid attention to unifying the name and isolation methods of extracellular vesicles (EVs)^29^. EVs are classified in the latest research into two categories, namely, ectosomes and exosomes^30^. Ectosomes are vesicles (50 nm‐1 μm in diameter) directly derived from the plasma membrane that include microvesicles, microparticles, and large vesicles; whereas, exosomes are nanosized extracellular membrane vesicles of endosomal origin secreted by most cell types with a diameter of 40–160 nm (average diameter of 100 nm) and a density of 1.13–1.19 g/ml^31^. They are typically cup‐shaped and shuttle‐shaped vesicles wrapped by a double‐layer lipid membrane with an average thickness less than 5 nm^32^, ^33^.

**Fig. 1:**
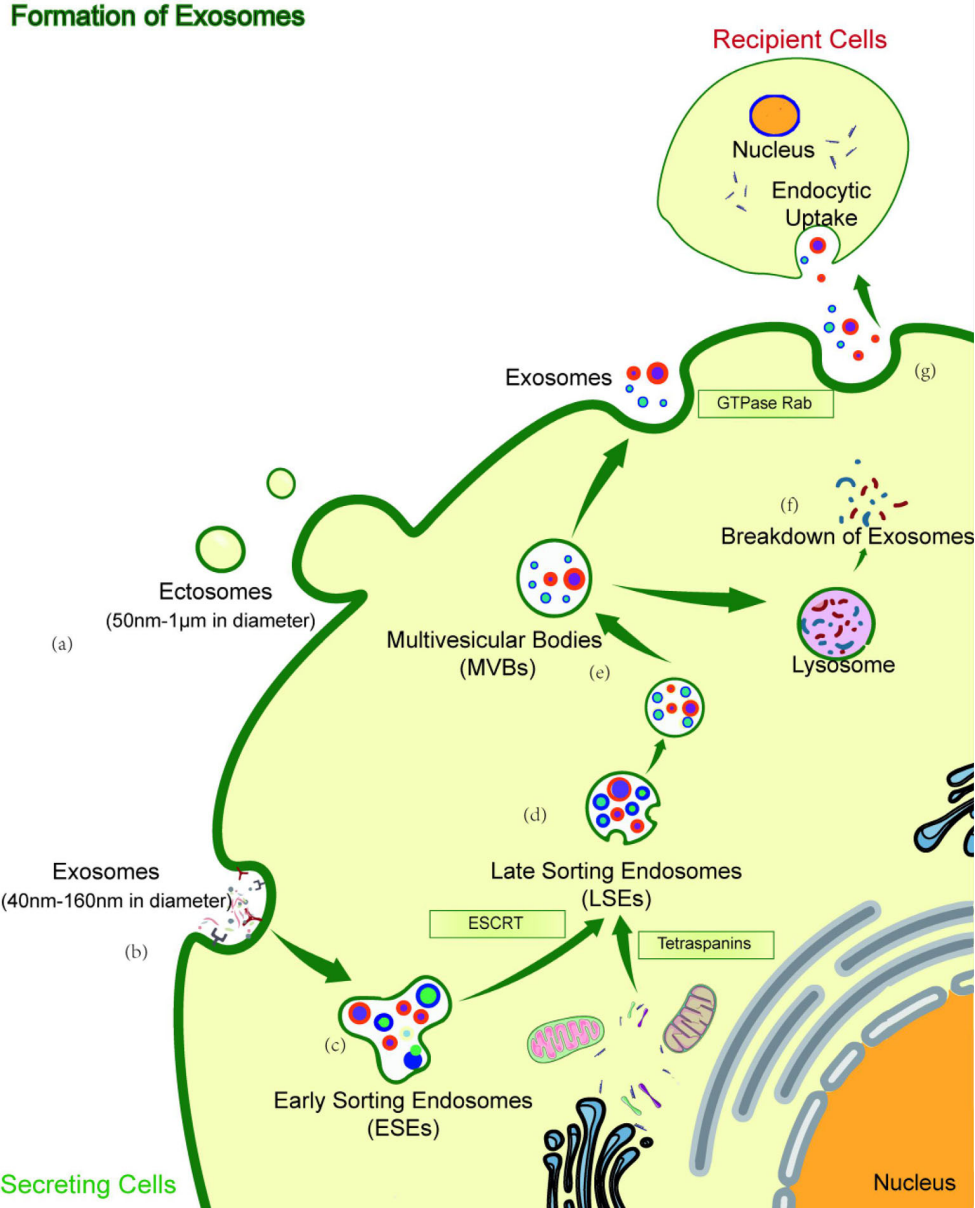
Biogenesis and identification of exosomes. (A) Identification of ectosomes and exosomes. (B) Endocytosis and plasma membrane invagination. (C) Formation of early sorting endosomes (ESEs). (D) ESEs give rise to late sorting endosomes(LSEs). (E) LSEs are gradually matured and transformed into multivesicular bodies (MVBs) containing exosomes. (F) MVBsare degraded by lysosomes. (G) The release of exosomes.

The production of exosomes is a special formation process. In brief, cytoskeleton proteins (such as actin and tubulin) interact with clathrin to form vesicles covered with clathrin through endocytosis and in vagination of the cell membrane. These vesicles after clathrin uncoating are known as early sorting endosomes (ESEs)^34^. Small molecules including proteins, mRNAs, and miRNAs derived from organelles such as endoplasmic reticulum, Golgi bodies, and mitochondria could be selectively transferred into early endosomes by two pathways, one of which is endosomal sorting complex required for transport (ESCRT)‐dependent and another one is ESCRT‐independent. The ESCRT‐independent pathway is mediated bytetraspanin membrane proteins (CD63, CD81, CD82, and CD9)and neutral sphingomyelinases 2 (nSMase2). Subsequently, reverse budding of early endosomes leads to the formation of intracellular vesicles enveloping small molecules, namely late sorting endosomes (LSEs)^35^. LSEs are gradually matured and transformed into multivesicular bodies (MVBs) containing exosomes. MVBs could not only be formed into autophagosomes or degraded by lysosomes but also be transported to the plasma membrane through the cytoskeleton and microtubule network. Depending on the Rab GTPase family, MVBs can release exosomes outside through exocytosis after fusion with the cell membrane^36^. During the formation process of exosomes, the specific endosomal proteins and some cellular contents are selectively sorted before the plasma membrane is sealed, forming the final contents of exosomes (Figure 1A‐G). The ongoing advancement of new technologies will also improve their classification. The classification of exosomes is particularly meaningful in biology as their production involves a unique intracellular regulatory process. Once secreted into the extracellular space, their composition and function are determined^37^.

### Heterogeneity and Markers of Exosomes

Exosomes reflecting the source of cells are highly heterogeneous. The heterogeneity of exosomes is manifested with their size, content, functional effect on the recipient cells. Different combinations of these characteristics lead to the complex heterogeneity of exosomes. Proteomic analysis of EVs potentially reveals the heterogeneity of exosomes and reflects the specific proteins derived from parental cells^38^. Previous studies have shown that proteomics has been widely used for investigations on the role of exosomes in inflammatory diseases to identify the proteins that are differentially expressed in disease and normal conditions. Meanwhile, specific proteins rich in exosomes are usually used as marker proteins, including tetraspanin membrane proteins (CD9, CD63, CD81, CD82, CD151, and Tspan8), specific stress proteins such as the heat shock protein (HSP) family, TSG101, endosome sorting complex required for the transport‐III (ESCRT‐3)‐binding protein ALIX, Rab GTPase family, cytoplasmic proteins, etc^39^. CD9, CD63, and CD81 are especially abundant in the exosomal membrane, and therefore frequently used for the identification of exosomes^40^.

### Exosomal miRNAs

The protein, mRNA, and miRNA composition of exosomes are dependent on the parental cell origin. Exosomes can transfer these cargoes to regulate the function of the recipient cell. These biologically active molecules perform a fundamental role in cellular communication and are also a targeted marker to assess the progression and treatment efficacy of disease^41^. Since exosomes are rich in resident mRNA and miRNA^42^, they treat diseases by carrying specific RNA molecules at the gene level. With the development of molecular biology techniques, researchers have gradually discovered the critical role of exosomal miRNAs in the progression of many diseases^43^.

### Exosomal miRNAs and Chronic Inflammation

Inflammation is defined as a preventive response to stimuli (such as pathogens or damages), the purpose of which is to eliminate stimuli, and to remove dead cells, inducing tissue repair. If controlled, beneficial outcomes are realized, otherwise, the consequences will be devastating if the stimuli persist or the inflammation has not been effectively resolved and become chronic. Typically, inflammation is classified as acute or chronic according to its intensity or duration. Exosome derived therapy emerges as one of the most advanced therapeutic strategies to modulate an overactive immune system. Immune cells actively secrete exosomes after noxious stimulation (such as pathogens or injuries). It has been reported that exosomes could act as either a promoter or inhibitor of inflammation, participating in immune regulation^44^, ^45^. The exosome‐based targeted therapy is a cell‐free treatment that is long‐term stability, and low or no immune response less toxic and produces fewer immune reactions^46^. There isincreasing concern for its role in the excessive inflammatory immune‐mediated response. Previous studies have evaluated the role of exosomal miRNAs in chronic inflammatory bowel disease (IBD)^47^, sepsis^48^, arthritis,^49^, diabetes^50^, atherosclerosis^51^, and neurodegenerative disease^52^, which suggests exosomes may potentially participate in the development of chronic inflammatory diseases.

### Exosomal miRNAs and Chronic Inflammation in Skeletal Muscle

Following skeletal muscle injury, macrophages will be activated to polarize and a variety of cytokines are released to result in the inflammatory response. Regarding the process of inflammation after skeletal muscle injury, acute inflammatory damage has long been concerned while chronic inflammatory damage is ignored. In fact, injury^53^, dystrophy^54^, and aging^55^ of skeletal muscle are accompanied by a state of low‐level chronic inflammation, pro‐inflammatory M1 macrophages were recruited into pathological muscle tissues, which shows the hidden dangers. Chronic inflammation is also a crucial cause of many diseases and complications, such as inflammation‐cancer transformation^56^, ^57^, type 2 diabetes^58^, dementia^59^ and sarcopenia^60^. Currently, it is beneficial to use anti‐inflammatory agents in the treatment of type 2 diabetes^61^. Long‐term sustained chronic inflammation can induce degeneration of skeletal muscle and related organs, showing weakened muscle energy and muscular function in mild cases, and connective tissue excessive accumulation in severe cases which is manifested with severe pathological reactions. As chronic inflammation further impairs the normal regeneration and repair process after skeletal muscle injury, the skeletal muscle cannot be fully repaired^62^, ^63^.

Current research on the inflammation after skeletal muscle injury mainly focuses on examining the implication of a single cytokine in skeletal muscle damage. Different respective contents of exosomes can be optimized for treatment at different times of skeletal muscle injury. Although it is the first important step in the investigation of mechanisms underlying inflammation in skeletal muscle, the comprehensive roles of selected cytokines (such as pro‐inflammatory factors) may not be predictable in a more complex *in vivo* environment, because dozens of regulatory proteins and their receptors fluctuate rapidly *in vivo*. Therefore, more critical information about inflammatory cells and their specific inflammatory factors in the process of skeletal muscle regeneration and repair is needed. Exosomes have gradually emerged as messengers of this key information on muscle damage^64^, ^65^, ^66^. They could mediate intercellular communication to produce various biological effects and high specificity of targeting.

## Exosomal miR‐223 May Be a Key Target of Chronic Inflammation in Skeletal Muscle

### Exosomal miR‐223

Exosomes are cell‐to‐cell communication vesicles that transfer abundant miRNAs across long distances and ubiquitously exist in the circulation^67^, ^68^. Then miR‐223 is the most abundant miRNA in human peripheral blood microvesicles (exosomes, etc.). These microvesiclesmay be derived from peripheral blood mononuclear cells, the primary source of circulating exosomes^21^, ^22^. MiR‐223 was first bioinformatically identified, then specifically expressed in the hematopoietic system. It can limit inflammation and prevent indirect damage during infection^26^, ^69^. Since persistently excessive expression of inflammatory factors may lead to chronic inflammation, miR‐223 is a negative feedback inhibitor that has the potential to adjust the inflammatory imbalance and accelerate the resolution of inflammatory processes^70^, ^71^. It can be seen that the research on the regulation of negative feedback factors in the inflammatory signaling pathway is essential in biology and medicine.

### Negative Feedback of miR‐223 in Chronic Inflammation

It is reported that miR‐223 can regulate neutrophil activity and enhance macrophage IL‐6 and IL‐1β production. Notably, miR‐223 plays a vital role in inhibiting the development of pro‐inflammatory cells^69^. Certified targets for miR‐223 that affect inflammation and infection include Pknox1^72^, granzyme B^73^, ^74^, IKKα^75^, Roquin^76^, STAT3^74^. Specifically, miR‐223 directly suppresses Pknox1 expression, which induces macrophage phenotype switch towards M2, as well as it attenuates NF‐κB‐mediated inflammation by targeting IKK‐α expression. miR‐223 can target Roquin (a negative regulator of IL‐17 production in lymphocytes) early in the myeloid lineage. Additionally, the up‐regulation of inducible miR‐223 reduces TLR‐triggered IL‐6 and IL‐1β production in macrophages by targeting STAT3 at the transcriptional level. For other immune cells, miR‐223 was down‐modulated, thereby up‐regulating its target gene, granzyme B, a significant component of cytotoxic T lymphocytes (CTLs) and NK cells granules. It is revealed that multiple functions of miR‐223 are associated with inhibition of many different target genes with specificity not merely used as a biomarker and addresses the indispensable role in negatively regulating the inflammatory process^77^.

A large body of evidence supports that miR‐223 is abnormally expressed in patient plasma in influenza^78^, chronic hepatitis B^79^, inflammatory bowel disease^80^, type 2 diabetes^81^, leukemic^82^, and lymphoma^83^.

### Negative Feedback Mechanism of Exosomal miR‐223 in Chronic Inflammation of Skeletal Muscle

Skeletal muscle macrophages participate in repair and regeneration following injury. Previous studies have reported that macrophages polarize into different phenotypes. It depends on which pathological mechanism of skeletal muscle injury is dominant. Exosomal MiR‐223 involved in the chronic inflammation of skeletal muscle injury^12^, ^84^. It could negatively regulate pro‐inflammatory factors such as TNF‐α^25^, ^26^, ^27^, inhibit inflammatory infiltration^28^, and eventually drive the ongoing inflammation to resolve.

An existing study has substantiated that the myeloid cells that initially invade into damaged muscle tissues are mainly neutrophils and pro‐inflammatory M1‐macrophages expressing iNOSat the early stages of chronic skeletal muscle inflammation, which clear away the damaged tissue^85^. As with acute muscle inflammatory injury, neutrophils and M1‐macrophages aggravate muscle injury through iNOS‐mediated arginine metabolism. However, differing from acute inflammatory injury, the influx of neutrophils and M1‐macrophages may be accompanied by the infiltration of M2‐macrophages which exhibit an M2a‐like phenotype during chronic inflammatory injury^85^. M2‐macrophages are characterized by elevation of IL‐4, IL‐10, CD206, and CD163 as well as the expression of arginases^86^. M2a‐like macrophages correlate with wound healing in other injured tissues and they can accelerate tissue repair and reduce inflammation. However, a pro‐inflammatory/anti‐inflammatory cytokine imbalance is found in chronic inflammatory injury, which may contribute to chronic condition.

**Fig. 2:**
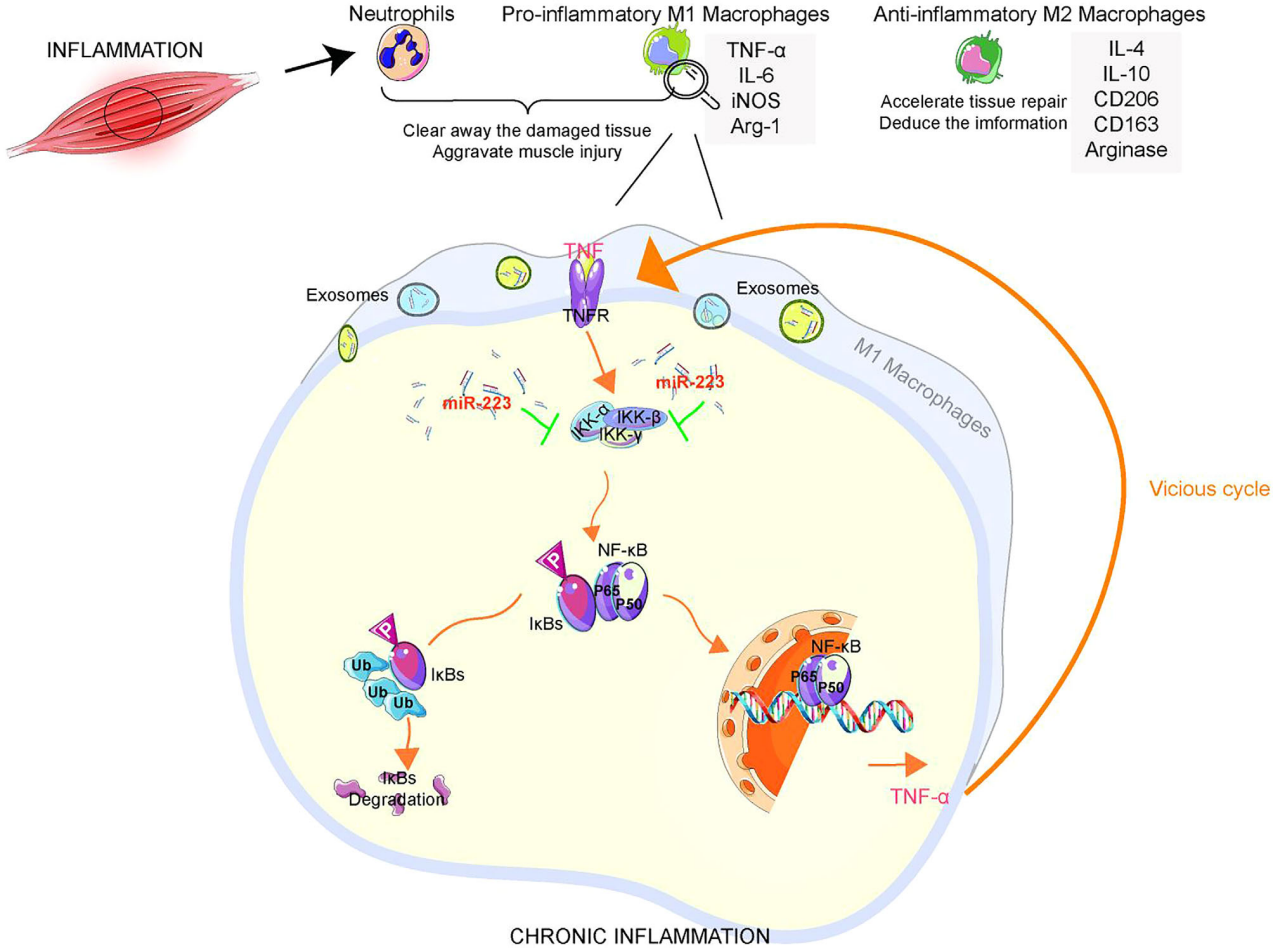
Exosomal miR‐223 is involved in the regulation of chronic inflammation in skeletal muscle. Neutrophils, and pro‐inflammatory M1‐macrophages first clear away the damaged tissue and aggravate the muscle injury, accompanied by the infiltration of M2‐macrophages. M1‐macrophages and M2‐macrophages release different inflammatory factors correspondingly. In general, NF‐κB binds to its inhibitory protein (IκBs) in the cytoplasm and remains in an inactive state. When IKK is activated, IκB‐α can be phosphorylated to remove its inhibition on NF‐κB, and allow free NF‐κB transferring into the nucleus to play the role of the transcription factor to produce TNF‐α. The long‐term presence of pro‐inflammatory TNF‐α continuously activates the NF‐κB signaling pathway, which forms a vicious circle, eventually leading to chronic inflammation. MiR‐223‐mediated inhibition of IKK‐α (a target of miR‐223) in macrophages, inhibits the activation of the NF‐κB inflammatory pathway and reduces the production of pro‐inflammatory cytokines such as TNF‐α, thus breaking the vicious circle of chronic inflammation.

An in‐depth investigation has revealed the presence of a series of key pro‐inflammatory factors, such as TNF‐α, in the regeneration and repair process after skeletal muscle injury. TNF‐α, a type of cytokine highly expressed by M1‐macrophages, exacerbates muscle damage. After an acute injury, the expression of TNF‐α in the muscle reaches its peak about 24 h after injury, which coincides with the time point when neutrophils and M1‐macrophages invade into the muscle tissues and induce damag.^87^. The pro‐inflammatory effect of TNF‐α is mainly attributed to activation of NF‐κB. In general, NF‐κB binds to its inhibitory protein (IκBs) in the cytoplasm and remains as an inactive state. When IKK is activated by TNF‐α stimulation, IκB‐α can be phosphorylated to remove its inhibition on NF‐κB, and allow free NF‐κB transferring into the nucleus to play the role of the transcription factor to produce TNF‐α. The long‐term presence of pro‐inflammatory TNF‐α continuously activates NF‐κB signaling pathway, which forms a vicious circle, eventually leading to chronic inflammation. It has been shown that exosome‐derived miR‐223 induces remission of chronic progressive inflammation. and maintains cellular homeostass.^26^, ^72^, ^88^, ^89^, ^90^. miR‐223‐mediated inhibition of IKK‐α (a target of miR‐223) in macrophages, inhibits the activation of the NF‐κB inflammatory pathway and reduces the production of pro‐inflammatory cytokines such as TNF‐α, thus breaking the vicious circle of chronic inflammation^65^, ^66^ (Fig. 2).

Cell experiments have also confirmed that exosomal miR‐223 could regulate the classical NF‐κB signaling pathway. NF‐κB remained in the cytoplasm in an inactive form after binding to the endogenous inhibitor of exosomal miR‐223. It is unraveled that miR‐223 can down‐regulate TNF‐α in the exosomes induced by inflammation, thereby significantly repressing the inflammatory response and reducing the area of muscle necrosis^31^, ^32^. miR‐223 has been demonstrated to negatively regulate NF‐κB activation and down‐regulate the expression of pro‐inflammatory factors such as NF‐κB, IL‐1β and IL‐6 in macrophages, potentially reducing the development of chronic inflammatory responses to skeletal muscle injury^12^, ^91^, ^92^. It follows that overexpression of miR‐223 decreases the level of cytokines by targeting corresponding genes to curb the excessive inflammation, making it possible to a positive transition from out‐of‐control to in‐control.

Meanwhile, animal experiments confirmed this relationship between exosomal miR‐223 and inflammatory damage as well^93^. For instance, miR‐223 can enhance the wound healing of mice after infection with *Staphylococcus aureus*. Additionally, restoration of miR‐223 in miR‐223‐deficient (miR‐223‐/Y) neutrophils at the wound potentially improves wound healing, indicating that miR‐223 may guide wound healing in a cell‐autonomous and non‐cell‐autonomous manner, and miR‐223 may inhibit the NF‐κB inflammatory signaling pathway in mouse epithelial cells^94^.

## Conclusions

Exosomes harbor nucleic acids, proteins, lipids and metabolites, making them not only important in cell communication, but also a replacement therapy to treating inflammation. Meanwhile, exosomes are regarded as “the third wagon (secondary to Circulation Tumor Cell (CTC) and Circulating Tumor DNA (ctDNA))” in the field of liquid biopsy secondary to ctDNA and CTC, which could be used as biomarkers of disease progression and treatment efficacy. MiR‐223 is the most abundant miRNA in human peripheral blood microvesicles (exosomes, etc.). As an anti‐inflammatory miRNA of chronic inflammation in skeletal muscle, it is negatively correlated with pro‐inflammatory factors such as TNF‐α in serum. Exosomal miR‐223 also plays an important role in the dysregulated inflammation of skeletal muscle and is expected to be a therapeutic target for chronic inflammation in skeletal muscle.

Additionally, miR‐223 is a negative feedback regulator with potentials to address complex chronic condition and is therapeutically viable. Therefore, Further research on more convincing experimental proof on the biological function of miR‐223 to improve clinical efficacy is urgently required. With the progress of molecular biotechnology in the future, it is also still a challenge to accurately regulate the effect of exosomal miR‐223 on target cells.

## Conflict of Interest

The authors declare that they have no conflicts of interest.
